# Effect of *Porphyromonas gingivalis* Infection on Healing of Skeletal Muscle Injury: An In Vivo Study

**DOI:** 10.3390/dj12110346

**Published:** 2024-10-30

**Authors:** Shintaro Shimizu, Kairi Hayashi, Yasuo Takeuchi, Gen Tanabe, Hiroshi Churei, Hiroaki Kobayashi, Toshiaki Ueno, Kenji Fueki

**Affiliations:** 1 Department of Masticatory Function and Health Science, Graduate School of Medical and Dental Sciences, Institute of Science Tokyo, Tokyo 113-8549, Japan; s.shimizu.spmd@tmd.ac.jp (S.S.); chu.spmd@tmd.ac.jp (H.C.); kunfu.rpro@tmd.ac.jp (K.F.); 2 Division of Sports Dentistry of Sports Science Organization, Institute of Science Tokyo, Tokyo 113-8549, Japan; 3 Department of Lifetime Oral Health Care Science, Graduate School of Medical and Dental Sciences, Institute of Science Tokyo, Tokyo 113-8549, Japan; takeuchi.peri@tmd.ac.jp; 4 Department of Sports Dentistry, School of Dentistry, Meikai University, Saitama 350-0283, Japan; gen.spmd@dent.meikai.ac.jp (G.T.); tueno@dent.meikai.ac.jp (T.U.); 5 Department of Periodontology, Graduate School of Medical and Dental Science, Institute of Science Tokyo, Tokyo 113-8549, Japan; h-kobayashi.peri@tmd.ac.jp

**Keywords:** *P. gingivalis*, periodontitis, motion analysis, skeletal muscle injury, satellite cell

## Abstract

**Background/Objectives:***Porphyromonas gingivalis* infection has been associated with various systemic diseases and may cause delayed healing of muscle injury. However, the relationship between muscle injury healing and *P. gingivalis* infection remains unclear. Our hypothesis was that *P. gingivalis* infection delays the healing of muscle injuries. **Methods:** Fifty-six 8-week-old male Wistar rats were randomly divided into two groups: sonicated *P. gingivalis* was intraperitoneally administered in one group (PG group), whereas saline was administered in the other group (CO group). Skeletal muscle injury was induced via cardiotoxin injections in all animals. The cross-sectional area of regenerating muscle cells was evaluated by haematoxylin–eosin staining, and the degree of muscle fibrosis was evaluated by Masson’s trichrome staining. The expression of paired box protein (Pax7) and myoblast determination protein (MyoD) and the identified stages of myocyte regeneration were analysed by immunohistochemical staining. Motion analysis was performed during walking. **Results:** The cross-sectional area of muscle cells was significantly smaller in the PG group on days 7 and 14 post-injury than in the CO group. The Pax7+/MyoD− ratio was significantly lower in the PG group on day 1 post-injury than in the CO group. Motion analysis of treadmill walking showed that the PG group had a lower minimum calcaneal height on days 3 and 7 post-injury than the CO group. **Conclusions:** This study suggests that administration of sonicated *P. gingivalis* in rats can delay the healing process of muscle injury. Further research is needed to understand this mechanism of delay of *P. gingivalis*.

## 1. Introduction

Skeletal muscle is the largest organ of the human body, accounting for 40–50% of body weight. Its functions range from exercise to protection, respiration, temperature regulation, and metabolism, and recent research has revealed its role as an endocrine organ [1]. Muscle injuries are characterised by slow healing and high recurrence rates. A study on the frequency of injuries sustained by athletes during the Rio de Janeiro Olympics reported that 65 of the 221 injuries were muscle injuries, and the most common injuries required seven or more days to heal [2]. Muscle damage leads to long-term functional impairment. Thus, treatments aimed at facilitating return to sports, such as hyperbaric oxygen therapy [3] and LIPUS [4], have been developed. However, the number of hospitals with such treatment facilities is limited, and symptomatic treatment is commonly used because muscle damage is considered a self-healing disease [5]. Of concern, delayed healing of damaged muscles is known to cause sequelae, such as fibrosis [6,7]. Older individuals in whom injury takes time to heal can develop locomotive syndrome as a result of resting and waiting for natural healing. Therefore, it is essential to elucidate factors that can inhibit recovery from muscle injury in daily life.

Patients’ general condition appears to significantly influence healing of skeletal muscle injuries. For example, systemic inflammatory conditions, such as age-related chronic elevation of inflammatory factors, chronic obstructive pulmonary disease, and chronic kidney disease [8,9,10], cause delayed muscle injury healing. The progression of the muscle regeneration process can be identified using molecular markers expressed in muscle satellite cells, such as paired box protein (PAX7) expressed at the periphery of muscle cells. Satellite cells are rapidly activated when muscle regeneration is required, such as after an injury. These cells then express myoblast determination protein (MyoD) and differentiate into myoblasts, which divide repeatedly and grow into muscle fibres [11]. The number of PAX7 and MyoD expressed around muscle satellite cells and the ratio to all nuclei are used as a method to estimate the stage of healing.

We hypothesised that infection with *Porphyromonas gingivalis* (*P. gingivalis*), a representative periodontal pathogen, delays the healing of muscle injuries. Periodontal disease not only causes deterioration of oral function by accelerating the destruction of the tissues that support the teeth but also affects distant organs. *P. gingivalis* and its constituents enter the bloodstream through periodontal pockets and circulate throughout the body. *P. gingivalis* is not the only bacteria that causes periodontitis. However, as shown in previous research, associations with various diseases have been reported in models administered with *P. gingivalis* alone. Various systemic diseases such as diabetes, arteriosclerosis, rheumatoid arthritis, and Alzheimer’s disease have been reported to be associated with *P. gingivalis* infection [12,13,14,15,16].

It is claimed that periodontal disease-causing bacteria create chronic inflammatory conditions as a cause of adverse effects on various diseases. For example, proinflammatory cytokine levels, especially IL-6 levels, have been reported to be chronically elevated in patients with periodontal disease [17,18,19]. *P. gingivalis*, which is often found in patients with periodontal disease, is regarded as a causative bacterium of periodontal disease. This bacterium has many pathogenic factors and is thought to enter the body through periodontal pockets and spread throughout the body. This phenomenon may affect the skeletal muscle; however, the relationship between *P. gingivalis* infection and healing of skeletal muscle injuries is still unclear. Therefore, this study aimed to evaluate the relationship between *P. gingivalis* infection and skeletal muscle healing using motion and histological analyses in a rat model of *P. gingivalis* infection.

## 2. Materials and Methods

### 2.1. Animals

**Figure 1:**
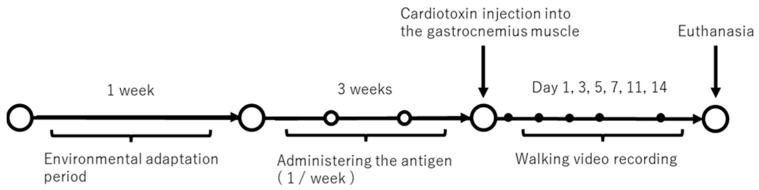
Experimental protocol.

Fifty-six 8-week-old male Wistar rats (Sankyo Labo Service Corporation Inc., Tokyo, Japan) were randomly divided into two groups: *P. gingivalis* (PG) and saline (CO). The rats were housed in standard cages and maintained in a 12 h light-dark cycle room. After a 1-week adaptation period, rats in the PG group received intraperitoneal administration of sonicated *P. gingivalis* weekly, whereas those in the CO group received administration of saline. After 3 weeks of administration, gastrocnemius injury was induced in the left leg of the rats via cardiotoxin (CTX) injections. Motion analysis was performed before injection and on days 1, 3, 5, 7, 11, and 14 after the injury. After the last motion analysis, the animals were euthanised, and muscle evaluation was performed. The experimental protocol is shown in Figure 1. The occurrence of respiratory distress, symptoms of distress, difficulty in eating and drinking, significant weight loss, and chronic bleeding was set as humane endpoints, and the experiment was to be terminated if any of these symptoms appeared. Euthanasia was performed by carbon dioxide overdose, taking into consideration any pain the animals were suffering. The sample size was determined by the minimum number required to investigate the effect of *P. gingivalis* on muscle healing.

### 2.2. Induction of Experimental Infection by P. gingivalis Injection

*P. gingivalis* ATCC 33277 was cultured anaerobically in brain–heart infusion broth (supplemented with 5 mg/L of hemin and 50 μg/L of vitamin K1) at 35 °C for 2 days. The bacterial cells were counted using a bacterial counting chamber, and the concentration was adjusted to 10^9^ cells/mL. The solution was centrifuged (8000× *g*, 4 °C, 10 min), and the supernatant was removed. The pellet was resuspended in physiological saline and sonicated. Rats in the PG group received intraperitoneal injection of 1000 μL of saline containing sonicated *P. gingivalis* once weekly for 3 weeks. Sonication was used to disrupt bacteria and maintain a constant concentration of antigens within the cells. Rats in the CO group received the same amount of saline. This was based on the preparation of *P. gingivalis*-administered animal models in previous studies [20].

### 2.3. Skeletal Muscle Injury Model

Three weeks later, the rats were wounded via intramuscular injection of CTX (Naja pallida; LATOXAN, ZA Les Auréats, France) into the left gastrocnemius muscle. CTX is a snake venom synthesised by cobras, which selectively damages muscle fibres. This model creation method has high reproducibility [21,22]. An injury model was created by administering up to 500 μL of diluted 10 μM CTX to the central part of the gastrocnemius muscle of the left hind limb [23].

### 2.4. Motion Analysis

Walking motion analysis was conducted on days 1, 3, 5, 7, 11, and 14 post-injury. The rats’ hind limbs were shaved, and an anatomical landmark was marked on the calcaneus. Walking motions during the treadmill exercise were recorded using a camera (D3300; Nikon, Tokyo, Japan) at a shutter speed of 1/800, and consecutive sets of three steps in which the head did not move up or down were extracted three times and analysed. Analysis software (Kinovea version 0.9.4) was used to record the minimum calcaneal height from the ground during walking, which was compared over time. To habituate the rats to the test, they were allowed to exercise at 20 m/min for 10 min for 1 week before the test.

### 2.5. Cross-Sectional Area of the Repaired Muscle Cell

After the 3-week experimental period, gastrocnemius muscles were harvested and immediately fixed in 4% paraformaldehyde for 72 h. The fixed samples were embedded in paraffin and sectioned into 7-μm slices using a microtome (Microm HM325 Rotary Microtome; ThermoFisher Scientific, Waltham, MA, USA). The samples were stained with haematoxylin and eosin (H&E), and the slides were observed and recorded using a slide scanner (VS200; Olympus, Tokyo, Japan). Cells with nuclei in the middle of muscle fibres were defined as repaired muscle cells. Thirty fibres were randomly picked from each, and the cross-sectional area (CSA) was measured using ImageJ software (ImageJ ver.1.54i, National Institutes of Health, Bethesda, MD, USA).

### 2.6. Analysis of Fibrosis

Delayed healing has been shown to result in the formation of scar tissue within the injured muscle [24]. Masson’s trichrome staining was performed 14 days after the injury to confirm the degree of muscle fibrosis. Eight high-power fields of injured muscles from each group were extracted from the stained slides and recorded. The area of stained collagen fibres was measured and compared using ImageJ software (ImageJ ver.1.54i, National Institutes of Health, Bethesda, MD, USA). This staining procedure has been described in a previous report [25].

### 2.7. Immunohistochemistry

Immunofluorescence staining was performed for PAX7, MyoD, and laminin to investigate the functions of the satellite cells surrounding the injured muscles. Staining was performed as follows. Paraffin-embedded gastrocnemius muscles were cut into 4-μm-thick sections using a rotary microtome. After blocking with normal goat serum, the sections were incubated with primary antibodies (MyoD, rabbit monoclonal antibody, Abcam, Cambridge, UK; Pax7, mouse monoclonal antibody, Abcam; Laminin, rabbit polyclonal antibody, Abcam, Cambridge, UK). The cells were then washed thrice with phosphate buffered saline (PBS) for 5 min each. This was followed by incubation with secondary antibodies (goat anti-mouse IgG-Alexa Fluor 594 and goat anti-rabbit IgG-Alexa Fluor 488; Abcam, UK). Subsequently, the cells were washed with PBS and mounted in mounting solution containing DAPI (Fluoroshield Mounting Medium with DAPI; Abcam, Cambridge, UK). Positively stained cells were observed under a microscope (BZ-X700; Keyence, Osaka, Japan). Images were processed and analysed using analysis tool of this system (BZ-X700; Keyence, Osaka, Japan). Eight high-power fields (HPFs) were extracted from each group, and the numbers of PAX7+/MyoD−, PAX7+/MyoD+, and PAX7−/MyoD+ plus DAPI-positive cells were recorded.

### 2.8. Statistical Analysis

Data are expressed as mean ± standard error (SE). Statistical differences between the two groups were analysed using the Mann–Whitney U test after testing for normality using the Shapiro–Wilk test. All statistical analyses were performed using R (version.4.2.2 Foundation for Statistical Computing, Vienna, Austria), and *p* < 0.05 was considered significant.

## 3. Results

### 3.1. Motion Analysis

**Figure 2:**
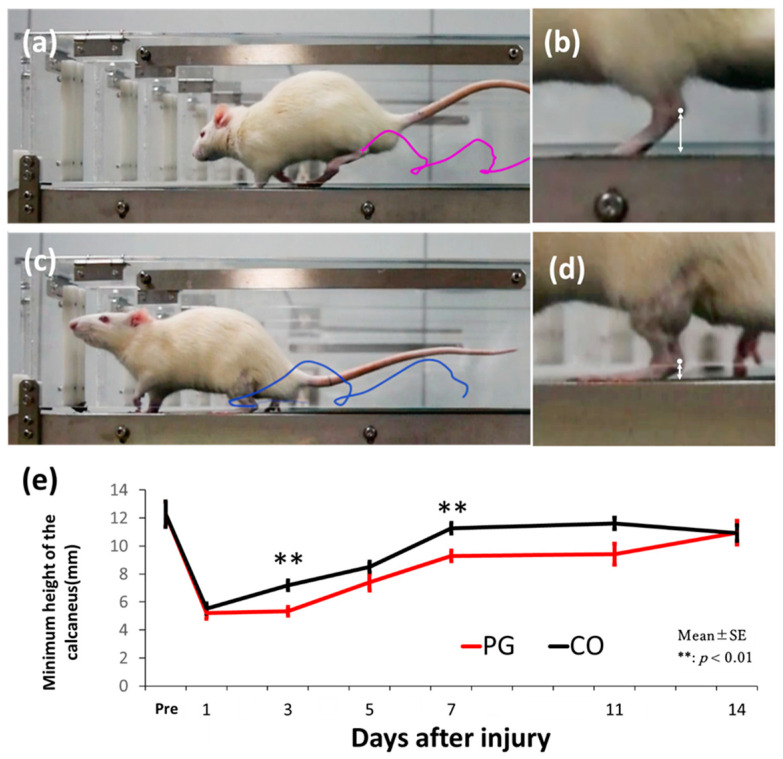
Walking motion analysis. (**a**,**b**) Walking motion analysis before injury and the magnified image of the calcaneus. (**c**,**d**) Walking motion analysis 1 day after the injury and magnified image of the calcaneus. White arrows indicate the measured heights. The minimum calcaneus height is significantly reduced after the injury. (**e**) Change in the minimum calcaneus height from before the injury to 14 days after injury. At 3 and 7 days after the injury, both the *P. gingivalis* (PG) and saline (CO) groups were confirmed to show recovery compared to the first day, but the degree of recovery was significantly greater in the CO group than in the PG group (*p* < 0.01).

Motion analysis and minimum height of the calcaneus are shown in Figure 2. No significant difference was noted in the height of the calcaneus between the PG (12.278 ± 0.911 mm) and CO (12.279 ± 0.880 mm) groups pre-injury (*p* = 0.977). A reduction in the height of the calcaneus was observed in both groups of animals 1 day after injury, but no significant difference was noted (*p* = 0.375). The height of the calcaneus was significantly smaller in the PG (5.338 ± 0.333 mm) group than in the CO (7.194 ± 0.323 mm) group at 3 days (*p* < 0.01) and 7 days after injury (*p* = 0.01). At 14 days after injury, both the groups showed further recovery, and almost the same minimum height of the calcaneus as that before injury.

### 3.2. Analysis of the Muscle Cross-Sectional Area

**Figure 3:**
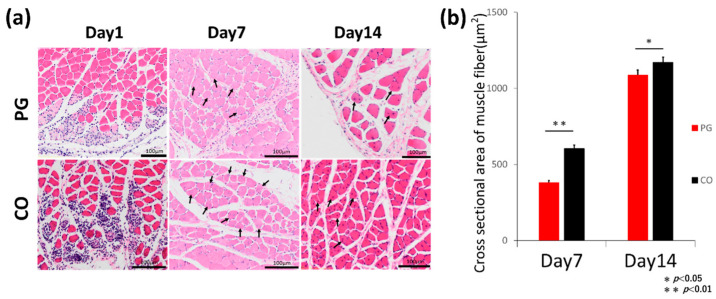
Stained images after injury. (**a**) Haematoxylin and eosin (H-E) staining images 1, 7, and 14 days after injury. On the first day after the injury, it can be observed that inflammatory cells are gathering in the injured area. On the 7th day, a regenerating muscle fibre with a central nucleus can be seen (arrows). On the 14th day, the diameter of the regenerating muscle fibres is visibly larger, indicating progressive regeneration (arrows). (**b**) Cross-sectional area of the regenerating muscle fibres 7 and 14 days after injury. The cross-sectional area of the regenerating muscle fibres was larger in the saline (CO) group at both day 7 and 14 after the injury.

Histological images at 1, 7, and 14 days after injury are shown in Figure 3. At 1 day after the injury, necrotic myocytes and infiltrating inflammatory cells were observed in the PG and CO groups. In both the groups, inflammatory cell infiltration was less on day 7 than on day 1, and muscle fibres with a nucleus at the centre of the injured area were observed. This indicated that the muscle cells had started to regenerate. On the 14th day, almost no inflammatory cells were found in the injured area, with progressive muscle cell regeneration. The CSA of the injured muscle fibres recovered to the same extent as did the undamaged portion; however, gaps were observed between the muscle cells. The CSAs of the repaired muscle cells between both the groups were significantly different at 7 and 14 days after injury. The cross-sectional area of the repaired muscle cells in the PG group was smaller than that in the CO groups at 7 days (*p* < 0.01) and 14 days after the injury (*p* = 0.019).

### 3.3. Analysis of Fibrosis

**Figure 4:**
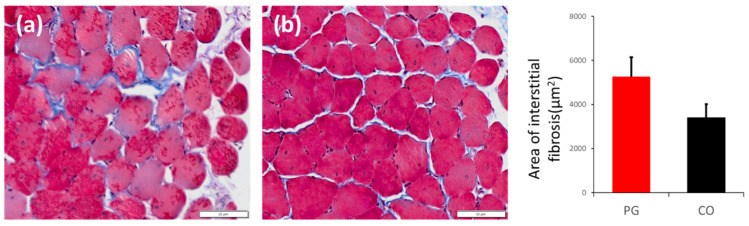
Histology of Masson’s trichrome staining of the *P. gingivalis* (PG) (**a**) and saline (CO) (**b**) groups 14 days after the injury. Collagen fibres are stained around a regenerating muscle fibre with a central nucleus Fibrosis occurs during regeneration. Comparison of the fibrosis area in a high magnification field.

Masson’s trichrome staining was used to compare the degree of fibrosis 14 days after injury. Fibrosis was observed in both the PG and CO groups (Figure 4). The PG group (5248.243 ± 888.709 μm^2^) tended to have a larger fibrosis area than the CO group (3400.014 ± 609.788 μm^2^), but no significant difference was observed (*p* = 0.161).

### 3.4. Assessment of Muscle Satellite Cell Activation

**Figure 5:**
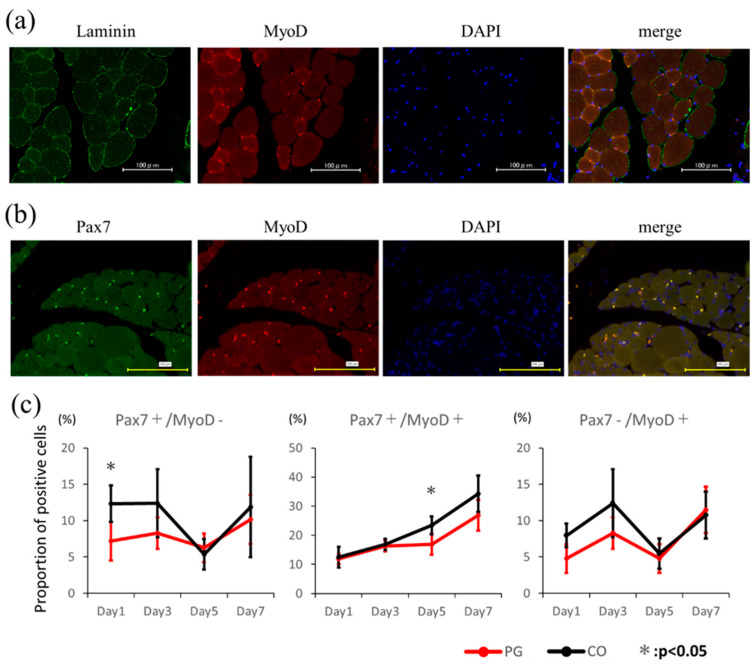
Immunostaining histology of laminin/MyoD/DAPI (**a**) and Pax7/MyoD/DAPI (**b**) 7 days after the injury. Satellite cells are located outside the sarcolemma and beneath the basement membrane of the muscle fibres. (**c**) Ratio of DAPI-positive cells 1, 3, 5, and 7 days after injury. Muscle satellite cells express Pax7 (+) MyoD (−) in the quiescent phase of activity and change to Pax7 (+) MyoD (+) and Pax7 (−) MyoD (+) as muscle regeneration progresses. A higher percentage of Pax7 (+) MyoD (−) on day 1 (*p* = 0.0379) and a higher percentage of Pax7 (+) MyoD (+) on day 5 (*p* = 0.0499) in the saline (CO) group suggest that the healing progresses faster.

Figure 5 shows immunostaining results and ratios of each positive cell type. Images stained simultaneously with laminin and PAX7 confirmed that the satellite cells were located outside the sarcolemma and beneath the basement membrane of the muscle fibres. The percentage of PAX7+/MyoD− cells was significantly higher in the CO (12.325% ± 2.490%) group than in the PG (7.172% ± 2.660%) group 1 day after injury (*p* = 0.0379). In addition, the percentage of PAX7+/MyoD+ cells was higher in the CO group 5 days after injury (*p* = 0.0499).

## 4. Discussion

In this study, we investigated the influence of *P. gingivalis* on the healing of muscle injury using a *P. gingivalis*-infected model with motion analysis and histological evaluation. Administration of sonicated *P. gingivalis* is advantageous because a constant concentration can easily be maintained. Further, it has been reported to increase the anti-*P. gingivalis* antibody titre in the blood when administrated in animals [26]; this method can be used to create a *P. gingivalis* model.

A previous study reported that the administration of sonicated *P. gingivalis* reduces endurance in rats [27]. However, there are few studies on the effects of bacterial administration on muscle function. Therefore, this study evaluated recovery of muscle function after injury. In a rat gastrocnemius muscle injury model, when a strong force is applied to the injured muscle upon landing, the injured rat cannot stand strongly. The minimum calcaneal height during walking decreased after injury and increased with recovery [28]. At 3 and 7 days after injury, the minimum calcaneal height in the PG group was significantly lower than that in the CO group, indicating that healing was significantly delayed in the PG group compared with that in the CO group. These results indicate that *P. gingivalis* injection has a negative effect on muscle injury healing in the early stages.

The results of histological analysis were consistent with those of motion analysis. Comparison of the cross-sectional areas of regenerating muscle fibres by H&E staining showed that the PG group healed more slowly than the CO group. In particular, 7 days after the injury, a significant difference between the PG and CO groups was observed. This suggests that delayed healing may occur during the early stages of wound healing.

In the case of muscle injury, delayed healing has been shown to cause fibrous tissue to enter the muscle cells, resulting in fibrosis. This fibrosis causes delayed healing as well as injury recurrence [29]. Muscle injury is characterised by a high recurrence rate, and early healing is important to prevent recurrence. Additionally, the fibres were stained with Masson’s trichrome to measure the degree of fibrosis. The PG group tended to have a larger fibrotic area than the CO group; however, the difference was not significant. However, the study period may have been too short to compare the extent of fibrosis. Further studies could allow for a longer recovery period to assess fibrosis, which may be related to the risk of recurrent muscle injury.

Immunostaining results showed a significantly higher proportion of PAX7+/MyoD− cells 1 day after injury in the CO group compared to the PG group (*p* = 0.0379). The number of PAX7+/MyoD+ cells was also higher in the CO group 5 days after injury (*p* = 0.0499). The number of PAX7+ /MyoD− cells increased in the early stages of muscle damage healing, and further progressively increased over several days, thereby promoting muscle damage healing. Immunostaining results suggested that wound healing proceeded earlier in the CO group than in the PG group. Higher rates of Pax+/MyoD− were observed earlier in the CO group, which may account for the earlier stages of healing.

Previous studies have shown that delayed healing of muscle wounds occurs during chronic inflammation [8], suggesting that the administration of *P. gingivalis* causes chronic inflammation. It has been clarified that *P. gingivalis* is easily transferred into the blood from periodontal pockets, especially in patients with periodontal disease. In periodontal disease model animals, the *P. gingivalis* antibody titres were increased in the blood, and *P. gingivalis* was also detected in distant organs such as the placenta [30] and brain [31]. Periodontal disease bacteria that migrate inside the body cause chronic systemic inflammatory conditions due to their specific toxins.

Chronic systemic inflammation may delay skeletal muscle injury healing by reducing satellite cell activity and interfering with the M1 to M2 shift of macrophages, which play an important role in the wound healing process. *P. gingivalis* infection is also known to cause vascular atheroma [32]. Peripheral ischaemia induced by atheroma is thought to adversely affect wound healing. It has also been reported that administration of *P. gingivalis* to animals inhibited normal glucose uptake in skeletal muscle [33]. Failure to perform normal metabolism can lead to delayed recovery when injured. Although there are several possible mechanisms for this delay, the mechanisms that cause delayed skeletal muscle wound healing remain to be elucidated. In addition, chronic inflammatory conditions are associated with IL-6 and TNF-α upregulation, which may have led to delayed healing. In this study, a significant difference was noted not only in histological evaluation but also in motion analysis, suggesting that recovery from muscle injury may be delayed in patients with periodontal disease. This research suggested that it is important to maintain a good oral environment to prevent colonisation of *P. gingivalis* bacteria for the rapid recovery of athletes from skeletal muscle damage caused by sports injuries. The result of this study provides an opportunity to improve the awareness of oral health among athletes and highlights the importance of oral health in preventing the deterioration of muscle function in older individuals.

This study also showed that fibrosis tended to increase with delayed healing. Inhibition of complete healing by fibrosis not only impedes the athletes’ return to play but also is associated with increased recurrence rates specific to the muscle injury.

One limitation of this study is that the model was established by the administration of sonicated *P. gingivalis* alone. Periodontal disease may not be caused by a single bacterial infection. Further research is needed to understand how *P. gingivalis* infection delays the healing of skeletal muscle injuries in the body.

## 5. Conclusions

There are two limitations to this study. One is that the model was established by administering only sonicated *P. gingivalis*. Periodontal disease may not be caused by a single bacterial infection. Further research is needed to understand how *P. gingivalis* infection delays the healing of skeletal muscle injuries in the body. In addition, this periodontal disease model was established by intraperitoneally administering sonicated *P. gingivalis*. Since periodontal disease bacteria spread from the oral cavity to the entire body, this model is thought to reproduce the systemic condition of periodontal disease patients. However, one of the limitations is that it is different from the actual infection route.

## Data Availability

The dataset can be accessed from the corresponding author upon reasonable request.
